# Psychomotor performances relevant for driving under the combined effect of ethanol and synthetic cannabinoids: A systematic review

**DOI:** 10.3389/fpsyt.2023.1131335

**Published:** 2023-02-24

**Authors:** Arianna Giorgetti, Vasco Orazietti, Francesco Paolo Busardò, Raffaele Giorgetti

**Affiliations:** ^1^Department of Medical and Surgical Sciences, Unit of Legal Medicine, University of Bologna, Bologna, Italy; ^2^Department of Excellence of Biomedical Science and Public Health, University "Politecnica delle Marche" of Ancona, Ancona, Italy

**Keywords:** ethanol, synthetic cannabinoids, psychomotor performances, review, systematic

## Abstract

**Objective:**

To determine whether the acute co-consumption of ethanol and synthetic cannabinoids (SCs) increases the risk of a motor vehicle collision and affects the psychomotor performances relevant for driving.

**Design:**

Systematic review of the literature.

**Data sources:**

Electronic searches were performed in two databases, unrestricted by year, with previously set method and criteria. Search, inclusion and data extraction were performed by two blind authors.

**Results:**

Twenty articles were included, amounting to 31 cases of SCs-ethanol co-consumption. The impairment of psychomotor functions varied widely between studies, ranging from no reported disabilities to severe unconsciousness. Overall, a dose-effect relationship could not be observed.

**Conclusion:**

Despite the biases and limitations of the literature studies, it seems likely that the co-consumption poses an increased risk for driving. The drugs might exert a synergistic effect on the central nervous system depression, as well as on aggressiveness and mood alterations. However, more research is needed on the topic.

## 1. Introduction

Originally marketed as “natural,” “safe,” and “legal” alternatives to cannabis, synthetic cannabinoid receptor agonists or synthetic cannabinoids (SCs) are one of the most widespread groups of New Psychoactive Substances (NPS). The class includes 224 compounds monitored, by the end of 2021, by the European Monitoring Centre for Drugs and Drug Addiction (EMCDDA) and 6,300 seizes amounting to 236 kg across Europe (1). International and national legislations strive to include these novel compounds within the lists of scheduled substances, but the process is limited by the dynamicity of the market (with approximately 15 SCs newly introduced only in 2021 (1)) as well as by several analytical challenges. Indeed, SCs are mostly not detected using regular rapid or screening tests and require target chromatographic techniques, which have to be constantly updated (2). The short detection window of many molecules in blood or serum makes it further challenging to detect SCs consumption, unless the presence of metabolites is ascertained. However, metabolites are often unknown and have to be studied *in vitro* or *in vivo* or both in order to prove the unequivocal consumption of SCs (3). These limitations are particularly relevant in driving under the influence of drugs (DUID) cases, where on-road screening analysis may not be efficient and the parent SC might have been completely metabolized by the time a driver is submitted to proper toxicological analyses. DUID constitutes an interaction between a driver and one or more psychoactive substances, whether licit or not. Mind-altering substances might affect different psychomotor functions which are relevant for driving, such as perception, attention, memory, judgment and evaluation, reasoning, decision-making, problem solving etc. Ethanol is the most important psychoactive substance detected in DUID cases and “drunk driving,” i.e., driving while being impaired by alcohol, represents a worldwide health issue (4). Acute consumption of cannabis is also associated with an increased risk of a motor vehicle crash, especially of fatal collisions, as shown by meta-analyses (5, 6). Moreover, since 1980, studies demonstrated that the impact of tetrahydrocannabinol (THC, the main psychoactive component of cannabis) on psychomotor function significantly increase in combination with ethanol intake, considering general performances, reaction speed factor, standing steadiness factor, and psychomotor coordination (7). Since THC and SCs both act on the cannabinoid receptor 1 and 2 (CB1 and CB2) located in the central nervous system (CNS), consumption of SCs can lead to performance deficits similar to or worse than those observed with cannabis use (8). As recently shown by a comparative analysis of animal and human data (9), psychomotor effects associated with the use of SCs have alarming implications for driving, including effects on attention, memory, response time, motor abilities, and interpretation of visual and auditory stimuli, as well as on behavior. However, scientific evidence on the topic is still limited, and data about DUID prevalence, traffic injuries, and fatal car accidents involving SCs is lacking. Moreover, it has to be considered that SCs usually show higher potency and possibly less predictable effects compared to THC, and this might be relevant for driving abilities, especially in combination with ethanol. We postulated that the combined intake of SCs and ethanol might affect the psychomotor performances to a greater extent than the drugs alone. The present study aims to provide a systematic scientific review of evidence focused on the effects, and particularly psychomotor function impairment, related to SCs and ethanol co-consumption.

## 2. Materials and methods

### 2.1. Literature search

A systematic literature review was performed in multiple databases (PubMed, Web of science) searching for psychomotor impairment related to co-consumption of SCs and ethanol. The review methods were established prior to conduct the research. The research question could be formulated as follows: P: exposed subjects; I: synthetic cannabinoids + ethanol; C: ethanol only or SCs only; O: effects on psychomotor functions relevant for driving. The following search terms were used, adapted for each database:

- ((((“Psychomotor Performance”[Mesh]) OR “psychomotor performance”) OR ((“Accidents, Traffic”[MeSH]) OR (“Motor Vehicles”[MeSH])) OR (impairment* OR accident* OR crash* OR collision*)) OR (intoxication* OR toxicity*))
- (((synthetic cannabinoids [MeSH Terms]) OR “synthetic cannabinoids” OR “synthetic marijuana” OR “synthetic cannabis” OR WIN OR JWH OR Spice))
- (ethanol OR alcohol OR EtOH)

No temporal limit was assigned to the literature research, which was restricted to the English language. Articles with the absence of toxicological findings (only reported consumption) were considered, as well as intoxication cases with co-consumption of the drugs. Death cases were included if both drugs were detected antemortem. Epidemiological studies reporting a rate of ethanol-SCs co-consumption were included but not actively targeted. Exclusion criteria were: off-topic results (e.g., no psychomotor performance described or assessed) (A), *in vitro* or animal studies (B), absence of both drugs in the evaluation of psychomotor performances (C), other (non-English language, non-availability of full-text) (D). The study selection was performed in duplicates by two blind independent authors (AG, VO). After noting the number of records, duplicates excluded, exclusion criteria were applied in a first selection to screen titles and abstracts of the retrieved hints. In order to achieve an inclusion as broad as possible, all articles admitted by at least one of the authors were considered for further steps. A second selection process was performed on full-texts, re-evaluating the same criteria and this time only articles selected by both authors were included. Literature search and assessment of eligible studies were performed according to the PRISMA Guidelines (10). The bibliography of the relevant articles was further cross-checked for inclusion of other articles.

### 2.2. Data extraction

From the included articles, the following information was extracted and used to build an Excel database:

- first author;
- year of publication;
- year of study. When not reported, it was approximated to the year of publication;
- country or place of study. When this was not available, the country of affiliation of the first author was used;
- type of article, classified into not mutually exclusive pre-defined categories: epidemiological study (prospective, retrospective, or cross-sectional), experimental study, case series, case report;
- sample size;
- type of population/case investigated, including SCs users/exposures, acute intoxications, criminal offenders, drug offenders, DUID, or death cases.

When considering epidemiological studies and case series, the rate of co-consumption of ethanol and SCs was reported or extracted. Particularly, for case series, the percentage of subjects positive for both drugs out of the total number of presented cases was calculated. In the evaluation of the case report/series which presented extractable data, the following data was additionally noted:

- epidemiology of the involved subjects, particularly age and sex;
- type of population/case;
- type of toxicological analysis performed, as reported in the article;
- latency between the offense or the SCs consumption and the toxicological analysis;
- number and semi-systematic name of the SCs involved, as well as blood/serum/plasma levels;
- blood alcohol concentration (BAC);
- other drugs eventually detected and their concentrations in blood/serum/plasma;
- psychomotor impairment as reported in the case.

Potential sources of conflict of interest, including funding, were additionally reported for each article. The evaluation of the Risk of Bias (RoB) was performed according to the tool for non-randomized studies of exposure (11). Data extraction was also performed by two authors (AG, VO) and a third author (FPB) was consulted in the case of disagreement.

## 3. Results

**Figure 1:**
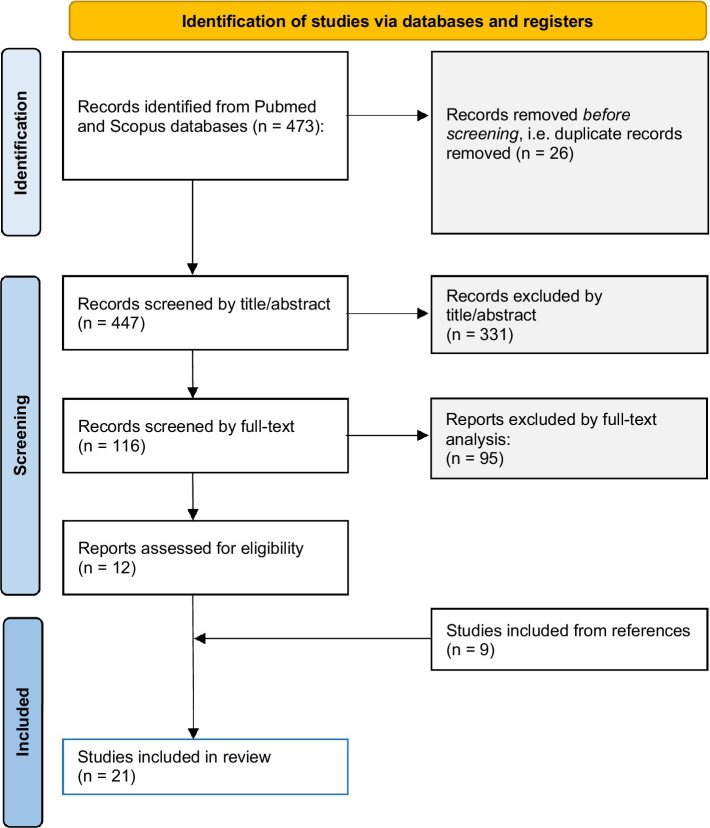
Prisma 2020 flow diagram, including database search, as applied for the present systematic review.

The literature search led to the identification of 473 hints, 447 duplicates excluded. After the selection process (Prisma flow chart, Figure 1 (10)) a total number of 20 (8, 12–30) were included. Studies were composed of *n* = 5 epidemiological studies (25%), *n* = 4 mixed epidemiological/case series (20%), *n* = 7 case series (35%), and *n* = 4 case reports (20%). Epidemiological studies were retrospective in three cases, prospective in two cases, cross-sectional in two cases, involved a retrospective analysis of prospectively acquired data in one case, while in one case it was not possible to establish this data. Articles were published from 2011 to 2022, with the oldest presented data pertaining to 2008–2011 and the newest approximately to 2020. The countries or places of study were: Australia, Germany, Hungary, Italy, Norway, Poland, Scotland (United Kingdom), Sweden, Turkey, and United States. United States and Germany provided the highest number of articles (6 and 4, respectively). A wide variety of sample size was seen, depending on the article type. In Table 1, detailed information extracted from articles, together with the calculated prevalence of ethanol and SCs co-consumption are shown.

**Table 1:** Prevalence of ethanol-SCs co-consumption as shown by epidemiological and non-epidemiological studies.

Year of study	Country/location	Type of article	Sample size	Type of population/case	Prevalence ethanol-SCs co-consumption	References	Funding/COI
2008–2011	Germany	Case series	29 re-analyzed	Acute intoxications	1/29 cases (3.45%)	Hermanns-Clausen et al. (2012) (12)	EU Commission funding. No COI
2010	Germany	Epidemiological retrospective+ case series	224 samples re-analyzed, 12 cases described	Criminal offenders and DUID cases	4/12 cases (28.6%)	Jaenicke et al. (2014) (13)	No funding. No COI
n.r. approximated to 2011	United States	Case series	11 cases	SCs adolescent users	10/11 (90.9%) cases	Castellanos et al. (2011) (14)	No funding. No COI
2010	United States	Epidemiological retrospective	572 calls, 464 exposures to SCs	Exposure to SCs	22/464 cases (4.7%)	Forrester et al. (2011) (15)	No funding. No COI
n.r., approximated to 2011	United States	Case report	1 case	Acute intoxication	–	Lapoint et al. (2011) (16)	No funding. No COI
2011	All world	Epidemiological cross-sectional	15,200 responses, 980 SCs users (use in the last year)	SCs users	90.5% of respondents used alcohol in the last month	Winstock and Barratt (2013) (17)	Self-funding and funding by the National Drug Research Institute. No COI
2011–2012	Australia	Epidemiological cross-sectional	316 online questionnaires	SCs users	77% of respondents used alcohol in the last month	Barratt et al. (2013) (18)	Funding by the National Drug Research Institute. No COI
2011–2012	Norway	Epidemiological prospective+ case series	726 cases analyzed, 16 positives to SCs	DUID cases and drug offenders	1/16 (6.3%)	Tuv et al. (2014) (19)	No funding. No COI.
n.r., approximated to 2012	United States	Case report	1 case	Acute intoxications	–	Pant et al. (2012) (20)	No funding. No COI.
n.r., approximated to 2013	Sweden	Epidemiological + case series	3,078 samples analyzed, 28% positives for SCs	Drug offenders and DUID cases	1/8 cases (12.5%)	Kronstrand et al. (2013) (21)	No funding. No COI.
n.r., approximated to 2013	Germany	Case series	7 cases	DUID cases	1/7 cases (14.3%)	Musshoff et al. (2014) (8)	No funding. No COI.
2014	Poland	Case report	1 case	Acute intoxication	–	Adamowicz. (2016) (22)	No funding. No COI.
n.r., approximated to 2015	Italy	Case report	1 case	Acute intoxication	–	Bertol et al. (2015) (23)	No funding. No COI.
2015	United States	Epidemiological retrospective	3,572 calls	Exposure to SCs	144/3,572 (4%)	Law et al. (2015) (24)	National Poison Data System, United States
2015	Turkey	Case series	16 cases	Adolescent acute intoxications	4/16 (25%) cases	Besli et al. (2014) (25)	No funding. No COI.
2015	Scotland (United Kingdom)	Case series	43 cases analyzed, 11 positives for SCs	Intoxications and death cases	5/9 intoxications (55.6%), 6/11 considering post-mortem cases (54.5%)	Seywright et al. (2016) (26)	No funding. No COI.
2015	United States	Case series	11 cases	Acute intoxications	1/11 (9.1%)	Katz et al. (2016) (27)	No funding. No COI.
2018–2019	Hungary	Epidemiological prospective + case series	116 cases analyzed, 48 positives for SCs	Acute intoxications	8/48 (16.7%)	Institóris et al. (2022) (28)	Funding by Ministry of Human Capacities, by EU-funded Hungarian grant. No COI
2016–2018	Hungary	Epidemiological prospective analyses, retrospective evaluation	2,639 cases	DUID cases	–	Institóris et al. (2022) (29)	No funding. No COI.
n.r., approximated to 2020	Germany	Case series	12 cases	Criminal offenders, DUID and death cases	1/3 (33.3%) DUID, 6/12 (50%) considering all cases	Kleis et al. (2020) (30)	No funding. No COI.

DUID, driving under the influence of drugs; n.r., not reported; SCs, synthetic cannabinoids; COI, conflict of interest.

The lowest prevalence of co-consumption was calculated as 3.45% in a case series of 29 intoxications (12), while the highest one, 90.9% was reported among adolescent users of SCs (14). Considering case reports/series involving SCs and ethanol presenting extractable data, 31 cases were included in the present systematic review and are shown in Table 2. Subjects were predominantly male (*n* = 29, 93.5%) with only *n* = 2 cases involving females (6.4%). The mean age was 29.8 years (standard deviation or SD = 10.4), ranging from 16 to 50 years. The type of population/case consisted of 20 acute intoxications, of which two were coupled to death cases, four criminal offenses, and seven DUID, one of which was coupled to a minor drug offense. Toxicological analyses consisted mostly of validated liquid chromatography (LC), high performance (HP), or ultra-high performance (UHP) LC, coupled to tandem mass spectrometry methods (MS/MS) with or without multiple reaction monitoring methods. In one case, the method was not reported (20) and in one case the results of the validation study were not shown (19). The latency between consumption of SCs (or offense) and toxicological analysis was the least reported information. Indeed, in 15 out of 31 cases (48.4%), this data was lacking. When reported, the latency spanned from slightly more than 30 min to a maximum of 8 h. The following synthetic cannabinoids were detected in combination with ethanol: JWH-122 (three cases), JWH-015, JWH-016, JWH-250, JWH-018 (three cases), JWH-201, JWH-307, AM-694, MDMB-CHMICA (six cases), 5F-AKB-48, MAB-CHMINACA, 5F-MDMB-PINACA (three cases), 5F-MDMB-PICA (nine cases), cumyl-CH-MEGACLONE, 4F-MDMB-BINACA (two cases), 5F-MDMB-P7AICA. Multiple SCs and co-consumption of ethanol were detected in 4 out of 31 cases (12.9%). In some cases, SCs were not quantified in blood/plasma/serum, but only qualitatively assessed (#2, #14, #15, #18, #26, and #30) or were identified in urine samples (cases #8, #12). Co-consumption of other drugs, besides SCs, and ethanol, was described in 13 cases (41.9%) and co-consumed drugs more frequently consisted of tetrahydrocannabinol (THC) or metabolites (8 out of 13). BAC ranged from 0.001 to 2.29 g/L. In two cases the alcohol consumption was reported by the subject but ethanol was not confirmed or quantified in blood (cases #9, #18). The impairment of psychomotor functions varied widely across cases, including the absence of abnormalities, nystagmus, light reaction delay, mood alterations until aggressiveness, increased/decreased vigilance, impaired motor coordination, impaired balance and gait, slurred speech, confusion, dissociative state, bradypsychia until unresponsiveness and coma. More details, including SCs concentrations, are shown in Table 2.

**Table 2:** Signs of psychomotor impairment and blood levels of SCs/ethanol in laboratory assessed co-consumption cases.

N.	Age	Sex	Type of population/case	Type of toxicological analysis	Latency	SCs blood/plasma levels (ng/ml) + BAC (g/L)	Other drugs detected mg/L	Psychomotor impairment	References
#1	22	M	Acute intoxication	Validated HPLC–MS/MS with MRM	n.r.	JWH-122 0.27 + BAC 1.03	–	Poisoning severity score 1 (minor drowsiness, vertigo ataxia, restlessness paresthesia mild visual or auditory disturbances, muscular pain, tenderness or mild cardiovascular, respiratory, gastrointestinal or metabolic symptoms)	Hermanns-Clausen et al. (2012) (12)
#2	46	M	Criminal offense	Validated LC–MS/MS with MRM	n.r.	JWH-015 < 0.19 + BAC 0.87	–	No abnormalities	Jaenicke et al. (2014) (13)
#3	19	M	DUID	Validated LC–MS/MS with MRM	n.r.	JWH-122 0.35 + BAC 1.13	Amphetamine 0.024	Inconspicuous mood	Jaenicke et al. (2014) (13)
#4	36	M	DUID	Validated LC–MS/MS with MRM	n.r.	JWH-016 < 0.33 + BAC 1.93	Cocaine 0.06, methadone 0.13, diazepam 0.95	Post-rotatory nystagmus (6 s.), unsteady gait, impairment of finger-to-finger and finger-to-nose test, retarded/delayed reaction, dizzy mind, confused talking, slurred speech	Jaenicke et al. (2014) (13)
#5	38	M	DUID	Validated LC–MS/MS with MRM	n.r.	JWH-250 2.88 + BAC 0.63	Tramadol 1.05, bromazepam 1.22	Post-rotatory nystagmus (10 s.), impairment of finger-to-finger and finger-to-nose test, depressive mood	Jaenicke et al. (2014) (13)
#6	48	M	Acute intoxication	Validated LC–MS/MS with MRM	Slightly >30 min	JWH-018 + BAC 0.038	–	Generalized seizure about 30 min after ingestion and recurrent afterwards	Lapoint et al. (2011) (16)
#7	29	M	DUID	LC–MS/MS (validation study n.r.)	1–2 h	JWH-122 0.5 + BAC 0.001	THC 10^−6^, LSD 10^−7^	Traffic accident and thus no clinical evaluation was performed	Tuv et al. (2014) (19)
#8	48	M	Acute intoxication	n.r.	n.r.	JWH-018 reported and confirmed in urine + BAC 1.4	–	Generalized tonic–clonic seizures, GCS 10/15	Pant et al. (2012) (20)
#9	20	M	DUID or minor drug offense	Validated LC–MS/MS with MRM	37 min	JWH-018 11 ng/g JWH-201 0.05 ng/g, ethanol reported but not confirmed	–	Severely intoxicated, with states of awakening alternated to unconsciousness, staggering, glossy eyes, incoherent and slurred speech	Kronstrand et al. (2013) (21)
#10	21	M	DUID	Validated LC–MS/MS with MRM	40 min	JWH-307 1.1 + BAC 1.74	–	Fast driving into a round- about, off-road into a ditch	Musshoff et al. (2014) (8)
#11	25	M	Acute intoxication + death	Validated LC–MS/MS with MRM	2–8 h	MDMB-CHMICA 5.6 + BAC 1.48	–	drunk and sleepy, had slurred speech, and it was also hard to communicate with him, then loss of consciousness, no reaction to light. Death after 4 days	Adamowicz. (2016) (22)
#12	25	M	Acute intoxication	Validated LC–MS/MS with MRM	6 h	AM-694 in urine + BAC 0.016	Midazolam 0.034	Agitation, hallucination, anxiety and paranoia after a major trauma	Bertol et al. (2015) (23)
#13	20	F	Acute intoxication	Validated LC–MS/MS with MRM	1 h	MDMB-CHMICA 5 + BAC 1.3	Diazepam 0.075	GCS 4/15	Seywright et al. (2016) (26)
#14	25	M	Acute intoxication	Validated LC–MS/MS with MRM	>3 h	MDMB-CHMICA <5, 5F-AKB-48 + BAC 0.8	11-nor-D9-THC- COOH 0.004	GCS 15/15	Seywright et al. (2016) (26)
#15	16	F	Acute intoxication	Validated LC–MS/MS with MRM	1 h	MDMB-CHMICA <1 + BAC 2.25	11-nor-D9-THC- COOH 0.009	GCS 14/15, acute behavioral disturbance with combativeness	Seywright et al. (2016) (26)
#16	18	M	Acute intoxication	Validated LC–MS/MS with MRM	40 min	MDMB-CHMICA 2 + BAC 2.29	–	GCS 7/15, serotonin toxicity (clonus, hyperreflexia), acute behavioral disturbance	Seywright et al. (2016) (26)
#17	20	M	Acute intoxication	Validated LC–MS/MS with MRM	2 h	MDMB-CHMICA 4 + BAC 0.79	Diazepam 0.28, desmethyldiazepam 0.34, 11-nor-D9-THC- COOH 0.023	Syncope, dissociative state, confusion	Seywright et al. (2016) (26)
#18	50	M	Acute intoxication	Unspecified LC–MS/MS	n.r.	MAB-CHMINACA + ethanol	–	Unresponsiveness	Katz et al. (2016) (27)
#19	32	M	Acute intoxication	Validated UHPLC–MS/MS	n.r.	5F-MDMB-PINACA 0.285 + BAC 2.27	–	Somnolence, vomiting, bradypsychia, slow pupillary reaction, hypoactive deep tendon reflex	Institóris et al. (2022) (28)
#20	28	M	Acute intoxication	Validated UHPLC–MS/MS	n.r.	5F-MDMB-PINACA 0.125 + BAC 0.69	–	Bradypsychia, nystagmus, conjunctival, slow pupillary reaction	Institóris et al. (2022) (28)
#21	32	M	Acute intoxication	Validated UHPLC–MS/MS	n.r.	5F-MDMB-PINACA 0.075 + BAC 2.43	–	Bradypsychia, slow pupillary reaction	Institóris et al. (2022) (28)
#22	36	M	Acute intoxication	Validated UHPLC–MS/MS	n.r.	5F-MDMB-PICA 8.21 + BAC 1.57	–	Unconsciousness GCS 13/15, confusion, bradypsychia, slurred speech, slow pupillary reaction	Institóris et al. (2022) (28)
#23	34	M	Acute intoxication	Validated UHPLC–MS/MS	n.r.	5F-MDMB-PICA 2.40 + BAC 1.08	–	Ataxia, bradypsychia, slurred speech, slow pupillary reaction	Institóris et al. (2022) (28)
#24	25	M	Acute intoxication	Validated UHPLC–MS/MS	n.r.	5F-MDMB-PICA 1.73 + BAC 0.41	–	Bradypsychia, slurred speech, ataxia, slow pupillary reaction	Institóris et al. (2022) (28)
#25	19	M	Acute intoxication	Validated UHPLC–MS/MS	n.r.	5F-MDMB-PICA 0.36 + BAC 1.22	–	Bradypsychia, GCS 13/15, slurred speech, slow pupillary reaction	Institóris et al. (2022) (28)
#26	33	M	Acute intoxication	Validated UHPLC–MS/MS	n.r.	Cumyl-CH-MEGACLONE not quantified + BAC 2.04	–	GCS 14/15, slurred speech, uncooperative, slow pupillary reaction	Institóris et al. (2022) (28)
#27	50	M	Acute intoxication + death	Validated LC–MS/MS with MRM	80 min	5F-MDMB-PICA 7, 4F-MDMB-BINACA 6.6+ BAC 0.25	Nordiazepam 0.022, THC 0.012, amphetamine 0.46	Suicidal ideation	Kleis et al. (2020) (30)
#28	38	M	DUID	Validated LC–MS/MS with MRM	150 min	5F-MDMB-PICA 0.89 + BAC 1.7	THC 0.0006	Multiple hit-and-run traffic accidents. Erratic behavior, confusion, slurred speech, severe balance deficiencies, delayed pupil light reaction	Kleis et al. (2020) (30)
#29	20	M	Criminal offense	Validated LC–MS/MS with MRM	90	5F-MDMB-PICA 0.11 + BAC 2.0	THC 0.006	Aggressiveness, slurred speech, logorrhea, subdued mood, severe staggering	Kleis et al. (2020) (30)
#30	22	M	Criminal offense	Validated LC–MS/MS with MRM	60 min	5F-MDMB-PICA 0.26, 4F-MDMB-BINACA 0.25, 5F-MDMB-P7AICA < 0.1 + BAC 2.1	THC 0.001, amphetamine 0.017	Aggressiveness, anxiety, irritability confusion, erratic thinking, delayed pupil light reaction	Kleis et al. (2020) (30)
#31	29	M	Criminal offense	Validated LC–MS/MS with MRM	50 min	5F-MDMB-PICA 2.5 + BAC 2.3	–	Changing moods with aggressiveness, increased vigilance, disorientation, persevering thinking; logorrhea	Kleis et al. (2020) (30)

Ref, reference; N., number of cases; M, male; F, female; LC, liquid chromatography; HPLC, high performance liquid chromatography; UHPLC, high performance liquid chromatography; MS/MS, tandem mass spectrometry methods; MRM, multiple reaction monitoring; BAC, blood alcohol concentration; LSD, lysergic acid diethylamide; THC, tetrahydrocannabinol; GCS, Glasgow coma scale.

Five of the retrieved studies received funding (12, 17), and none disclosed a conflict of interest. Considering the risk of driving impairment after co-consumption of SCs and ethanol as the numerical result assessed, some biases were found in the selected publications. The tolerance of subjects to the effects of SCs and ethanol could not be evaluated in the included articles, and this could act as a confounding factor. Similarly, other factors, e.g., the type of consumption (whether occasional or repeated, the administration way), the consumption of additional drugs, food intake, individual metabolic phenotype etc. could not be always evaluated. Regarding measurement errors in exposure, a risk of bias might arise from the delayed time between SCs and ethanol co-consumption (or offense) and toxicological analysis, as well from the absence of standardized sampling for SCs and of routine drug testing. Moreover, although the employed methodologies were mostly validated, some limitations have to be considered for analytical methods (13). It cannot be excluded that, due to novel substances being released on the market or due to active metabolites not detectable by the method, the measurement of the exposure was biased (26). As already reported, the selection of participants was different from one study to another, not only geographically but also regarding the type of consumption (e.g., naïve vs. SCs users, age groups etc.). As an example, cases from the emergency departments might lead to an overestimation of the risks (12). For cross-sectional studies, a bias connected to the possibility of participants taking the survey more than once from the same computer could not be excluded (17). Surveys and studies at poison centers (15) could be affected by a bias of recall and selective reporting (17, 18), which can alter both selection and outcome measurement (18). Finally, the measurement of the outcome might be biased by the use of proxy for driving disability, such as psychomotor impairment and acute intoxications.

## 4. Discussion

In drivers involved in accidents, toxicological analyses often reveal the presence of alcohol, cannabis as well as a combination of the two drugs. Despite the similarities in effects between cannabis and SCs, data regarding the prevalence of SCs in DUID cases and traffic accident is still scarce. As reported in past studies, the prevalence of diversion of SCs alone (not combined with other drugs) might range from 0.2 to 14%. The study of Jaenicke et al. (13) reported a prevalence of 2.8% of SCs in cases of traffic offenses, similar to what reported for Norwegian drivers (19). A much higher prevalence, approximately 30%, was reported among samples already suspected for SCs use by the police (21). The low availability of epidemiological studies limits the possibility of ascertaining the SCs-mediated risks of traffic accidents, which is not straightforward in the absence of controlled clinical studies and real-driving simulations (19). The prevalence of co-consumption of SCs and alcohol/drugs in this specific setting would be of paramount importance to establish which drugs and tests should be part of a standard drug analysis. Particularly, since SCs are not efficiently detected by immunological and screening methods and require target analyses, it would be beneficial to understand whether DUID cases involving SCs might be intercepted among drivers positive for alcohol or positive for other drugs usually detected at screenings. Furthermore, this data might allow comprehending whether the reason for using SCs resides in the low detectability at screening tests or whether it is based only or partially on other factors, e.g., low prices, easy availability, etc.

In order to understand the risk of traffic accidents resulting from a co-consumption of drugs, both pharmacokinetic and pharmacodynamic interactions should be taken into consideration. It is known that in the presence of ethanol, oral cocaine might be subjected to transesterification *via* carboxylesterases enzymes (CES) to form a new active metabolite, cocaethylene. A similar transesterification process has been recently described for several SCs by *in vitro* studies and confirmed in a single case (31–33). The generation of ethyl ester metabolites, although these compounds have shown low pharmacological activities compared to the parent SCs (31), might result in enhanced psychoactive effects (32) and this possibility has yet to be extensively explored *in vivo*. From a pharmacodynamic point of view, the high density of cannabinoid receptors located on the axons and terminals of the γ-aminobutyric acid (GABA)-ergic striatal neurons of the basal ganglia and the glutamatergic granule cells of the cerebellum suggests that SCs may play a modulatory role in the control of movements and motor functions. GABA receptors occupy a prominent role in the CNS effects mediated also by ethanol. The similarities in pharmacodynamics might represent some physiological basis to hypothesize an increased effect on CNS function when ethanol and SCs are co-ingested or co-consumed. Also, a possible interaction between SCs metabolites and the enzymes necessary for ethanol metabolism, i.e., alcohol dehydrogenase (ADH) and aldehyde dehydrogenase (ALDH), has been postulated. Indeed, it has been shown that a mono-hydroxylated metabolite of JWH-018, but not the parent compound, could act as a substrate for the two nicotinamide adenine dinucleotide (NAD(+))-dependent enzymes, competing for ethanol (34). To the best of the authors’ knowledge, only one study on animal evaluated the effects on motor impairment and glutamate neurotransmission of the combination of ethanol and SCs (JWH-018 and AB-CHMINACA) compared to ethanol or SCs alone. JWH-018 (0.05 and 0.1 mg/kg) and AB-CHMINACA (0.005 and 0.01 mg/kg) injected intraperitoneally 10 min before the administration of ethanol (2 g/kg) significantly enhanced ethanol-induced motor impairment, as shown by the latency to fall from an accelerating rotarod (35). Results were similar to the administration of tetrahydrocannabinol (THC), with a peak of effects 15 min after administration and a duration of 75 min. The effects were antagonized by CB_1_ selective antagonists, showing the relevance of the role of these receptors (35). Furthermore, the glutamate release in the cerebellum was studied, to check whether the neurotransmission in the cerebellum might play a role in motor impairment. Glutamate release was unaffected by the injection of SCs, but a decrease was noted after the co-administration of JWH-018 and ethanol, and this reduction was more marked when compared to the administration of ethanol alone (35). These results appear interesting, especially considering that BAC concentrations were not affected by treatment with SCs, so the impairment detected is likely not a result of a change in ethanol distribution. As in all kinds of animal studies and repeatedly mention by our group, caution must be paid not to directly suppose that the results on mice might be translated easily to humans (9, 36).

The co-consumption of multiple drugs poses several challenges in forensic toxicology, not only in terms of analytical determination, but also of interpretation. It might be difficult, especially when dealing with NPS, to assess the contribution of the drug to a detected impairment (19). Subsequently, this review was focused on the co-consumption of SCs, which have been recently shown to pose a risk to driving abilities, and ethanol, extensively studied in terms of driving impairment.

### 4.1. Prevalence of co-consumption studies

#### 4.1.1. Surveys and poisoning centers

It is well-known that the recreational use of SCs is often accompanied by alcohol intake (14, 25, 28, 37–39), which could involve up to 97% of synthetic cannabis users (17). Particularly, as shown by a survey performed on more than 1,000 individuals, binge alcohol drinking appears to be common in SCs users (40). A valuable source of information for estimating the prevalence of co-consumption might be represented by the data of poison centers. The Texas poison centers, in 2010, detected 464 calls related to exposure to SCs and among these 4.74% calls reported a polysubstance abuse in combination with alcohol (15). The percentage was confirmed by the United States National Poison Data System in 2015 (24). An online questionnaire submitted in Australia to 316 SCs users demonstrated that 77% of respondents had consumed ethanol in the last month, although this does not necessarily imply an acute exposure to both drugs (18). Higher co-consumption rates were reported in a large global survey, in which 90.5% of SCs users (who declared consumption of SCs in the last year) also reported use of alcohol in the last month (17). It is known that data from poisoning centers, not corroborated by analysis of blood samples, might be biased, and that questionnaires and survey might suffer from bias of recall and selective reporting. Thus, results must always be taken carefully. Notwithstanding these limitations, this kind of epidemiological cross-sectional data seems to point to a rather common pattern of co-consumption of ethanol and SCs in SCs users.

#### 4.1.2. Case series

Case series offer, in contrast to epidemiological studies, the opportunity to estimate the prevalence of co-consumption in more specific or hard-to-reach categories (e.g., intoxications vs. DUID cases), possibly leading to the emergence of different patterns of consumption across subpopulations. For example, case series might allow reaching sub-populations like adolescent SCs users, where the rate of co-consumption seems even higher (91%) than the general population. This result is consistent with the one reported globally by respondents with a mean age of 23 (14, 17). Case series might also allow a more in-depth study of the singular cases, coupled with toxicological analyses and confirmations, overcoming the limitations of circumstantial data. However, they are often characterized by a smaller sample size, which factor increases the likelihood of selection biases and further decreases the reliability of the prevalence estimation. Keeping this in mind, an extremely high rate of co-consumption of ethanol and SCs was noted in acute intoxications occurring in Scotland (55.6%) in 2015 (26). Conversely, only 3.5% of acute intoxications in Germany around 2008–2011 tested positive for both drugs (12), and only 9.1% of 11 cases reported by Katz et al. (27). In Turkey, co-consumption was estimated around 25% among adolescents with acute intoxications, but a higher percentage of subjects (66.7%) declared a chronic alcohol habit (25). The largest case series of the present review took place in Hungary, where 16.7% of 48 acute intoxications resulted positive for both SCs and ethanol (28). In a retrospective evaluation of traffic and criminal offenses cases in 2010, 422 serum samples were re-analyzed for SCs. The pattern of SCs consumption in traffic offenders seemed to be predominantly a multi-drug combination (13). The relatively high prevalence of combined consumption of ethanol (usually tested in DUID cases) and SCs suggests that the low detectability of drugs at routine drug test might be a minor reason sustaining the abuse of NPS, compared to the easy availability of SCs or to other factors (13). In Norway, all DUID and criminal cases involved the consumption of other psychoactive substances besides SCs, especially THC, amphetamines/methamphetamines, and benzodiazepines (BDZ). Only one case involving ethanol was detected. It appears likely that SCs-positive drivers do not differ from other drivers and are not predominantly pushed, in the choice of the substance, by the possibility of avoiding a positive drug screening test (19). However, considering the limitations already reported, more data and future research is needed on the topic. For the same reason, it is hard to define which SCs prevails, and this seems to be related to the availability and spreading on the NPS market. Regarding the characteristics of consumers, a strong prevalence of male drivers was seen. The combination of other drugs of abuse, which is common, might suggest that those who consume SCs and ethanol are usually quite experienced and not extremely young (19). Indeed, the mean age in the extracted articles was around 30. However, considering the focus on psychomotor performance, this result might be biased by the selection of DUID cases, which cannot involve younger subjects.

### 4.2. Psychomotor performances

Epidemiological studies rarely address the issue of psychomotor performances and effects related to the co-consumption of multiple drugs. However, some interesting data might occasionally emerge. Among 316 SCs users questioned online by Barratt et al. (18), those who concurrently consumed alcohol (in the last month) experienced statistically more severe effects. Indeed, based on self-reported effects, decreased motor co-ordination was reported by 38% of responders, together with dizziness (20%) dissociation (22%), confusion (18%), and slurred speech (14%), with a greater number of side-effects when co-consuming ethanol (18). It is also interesting to note that coma occurred in only 7 of the 464 patients who contacted the Texas poisoning center after a SCs consumption and 3 of these had co-consumed other substances including ethanol (15).

#### 4.2.1. JWH-type SCs and ethanol

Within the case series/reports here considered, authors reported five cases of traffic offenses in which a combination of SCs (of the JWH type) and ethanol was detected, describing effects from none to a severe coordination impairment (12, 13). In one criminal offense case (case #2), JWH-015 in concentrations below 0.2 ng/ml, coupled with a BAC of 0.87 g/L, did not produce any abnormality of psychomotor functions (13). JWH-015, however, is a rather “old” SC, which shows greater affinity to CB_2_ rather than to CB_1_ receptors. However, the latter is the most abundant in the CNS (41, 42). The role of the SC was also likely mild in case #4 where JWH-016, another naphthoylindole characterized by scarce pharmacological data (43), was detected in combination with high levels of ethanol and multiple co-consumed compounds (13). Indeed, the BAC of 1.93 g/L could well explain the coordination deficits and the cognitive impairment described, though a synergistic effect of low doses of SCs cannot be excluded. Similarly, in one case of erratic driving (case #10) with fast driving and running off the road reported by Musshoff, ethanol was assumed to be the main cause of unsafe driving, despite the detection of 1.1 ng/ml of JWH-307 (8). This compound has a high affinity (43) and acts like a full agonist at CB receptors in mice, leading to antinociception, hypothermia, catalepsy and suppression of locomotory activity (44). Among the 29 SCs-related intoxications reported by Hermanns-Clausen et al., one tested positive for JWH-122 and alcohol (case #1). The subject displayed mild symptoms, which are consistent with the effect of ethanol alone (1.03 g/L). In one DUID case (case #3) of co-consumption of JWH-122, amphetamines and ethanol (1.13 g/L), the resultant psychomotor effect, i.e., inconspicuous mood, could not be associated with certainty to ethanol or SCs and the authors concluded that the amphetamines likely played a role (13).This might be due to the low concentration and relatively low potency of JWH-122 (EC_50_ values twice compared to that of JWH-018 (45, 46)), or to a developed tolerance to the SCs effects. JWH-122 was also found in slightly higher concentrations in a case of traffic accident (case #7), coupled with very low BAC, low concentrations of THC and of LSD (19). In this case, contrarily to the previous one, the influence of other substances was likely low, given the low levels in blood. However, these concentration were approximately in the same range, so that some confounding factors (considering the same compound potency) likely have to be additionally considered, e.g., delay, synergic effect, tolerance, etc. In case #5, the low BAC (0.63 g/L) appears inadequate to produce the depressive mood and the impairment of coordination, most likely attributable to a relatively high concentration of JWH-250 in combination with tramadol and bromazepam, which are CNS depressants (13). JWH-018 is one of the most studied SCs, which was originally characterized due to its higher potency compared to THC (45). In the present study, it is interesting to note that it was associated to the development of generalized seizures (16, 20) in two cases (case #6 and case #8), in which no quantitation in blood was achieved. It is known that drinking acutely raises the threshold of seizures by acting as a CNS depressant, while reducing it after its cessation/withdrawal. In case #6, ethanol concentration was very low and a past consumption can be hypothesized, although the seizure started soon after the consumption of JWH-018. In case #8, BAC was high and should have theoretically protected the person from seizure. Generalized tonic–clonic seizures have been reported after the consumption of Spice products and this effect seems to be mediated by CB1-receptors (47–49). People affected by epilepsy are usually granted the driving license only after a seizure-free period and, depending on the legislation, they are evaluated by specific commissions (50). In the case of SCs consumption and especially of SCs and ethanol co-consumption, the risk for an unpredicted seizure in a previously seizure-free person has to be considered, especially when alcohol consumption precedes SCs intake.

Interestingly, JWH-018 was also detected in a case (case #9) of severe intoxication, in which the patient experienced periods of unconsciousness (21). The relatively high concentration detected (11 ng/g) is likely due to the low latency between consumption and sampling (only 37 min). Since latency is not reported in many other cases in the literature, although JWH-018 has been repeatedly detected in DUID cases, it is hard to compare this case to others (9). The severity of the clinical picture, however, was most likely connected to the SC, given the absence of confirmed BAC. It is well known that, by increasing the latency between consumption or traffic offense and toxicological analysis, the opportunity of detecting a co-consumption is reduced, especially when dealing with SCs, which usually display a small detection window in blood. The presence of cases in which one or both drugs were only reported or only found in urine further confirms the importance of a rapid sample collection (20, 21, 23). Urinary methods, based on the detection of SCs metabolites, might allow a greater detection window but would not allow to ascertain a psychoactive effect at the time of driving. Interestingly, in the literature collected, latency or delay of toxicological analysis was frequently not reported, and this represents a challenge for the assessment of the effect of drugs on psychomotor performances relevant for driving. The drug stability is another factor which should be considered when evaluating the latency between a driving offense and the toxicological analysis. Among the studies included in the present revision, a stability of SCs at 20°C for 4 days was proven by Jaenicke et al. (13). However, data on stability, even in the freezer, for very long periods is lacking and this appears as a limitation of those studies involving the re-analysis of samples stored for years (13).

#### 4.2.2. Valinate/tert-leucinate SCs and ethanol

Several cases of acute intoxication were found positive for ethanol in combination with MDMB-CHMICA, with or without concurrent SCs or substances (22, 26). MDMB-CHMICA was first reported around 2014 and showed high potency *in vitro*, 10 times higher than JWH-018 in similar assays (51, 52). Thus, it is not surprising that severe psychomotor impairment was observed in cases of co-consumption of MDMB-CHMICA and ethanol (e.g., case #11, #13), with death resulting from otherwise not comatose BAC levels (22, 26). Interestingly, the effects on the Glasgow coma level (GCS) might appear dose-related, with GCS around normality (14/15, case #15) at <1 ng/ml, 7/15 at 2 ng/ml (case #16) and GCS 4/15 when MDMB-CHMICA was around 5 ng/ml (case #13). However, the dose relationship is not respected when considering BAC, death cases or other psychomotor alterations (26). The typical detrimental effect described with the co-consumption of MDMB-CHMICA and alcohol is clearly represented by a CNS depression and this is not an unexpected finding. In literature cases, outside of this review, sudden collapse or unresponsivity has been reported when SCs and ethanol were co-consumed (53, 54), suggesting that CNS depression might be enhanced unexpectedly by the consumption of both drugs. Behavioral disturbances and serotonin toxicity have additionally been described (case #16), and it is unclear whether these could result from ethanol, from SCs or from both. Agitation, irritability, anxiety, and paranoia up to psychosis, commonly self-reported by SCs users, have been described in case reports/series and in multicenter, hospital-based registries (12, 16, 55–57). Changing in mood (aggressiveness, increased vigilance, or deflected mood) and affected cognition (errant thoughts, confusion, and disorientation) would likely have a negative effect on driving (30). In this context, the co-consumption of alcohol might increase the behavioral toxicity of SCs, acting through its disinhibitory effects. Similarly, the co-consumption of drugs might be responsible for the onset of a dissociative state (case #17), which was reported in a case of acute intoxication with MDMB-CHMICA, low ethanol and benzodiazepines levels (26). However, moderate doses of SCs, even with no co-consumed drugs, have been shown to induce psychotomimetic symptoms including dissociative effects (58, 59). On the other hand, alcoholic “blackouts” have been described as dissociative states. Unpredictable effects, but not relevant for driving, were reported in 2 of the 4 cases of co-consumption described by Besli et al. (25) consisting in bradycardia, although, since alcohol might also induce hypotension and ECG changes, the role of NPS could not be established. A number of cases involving 5F-MDMB-PICA (a SC with EC50 similar to JWH-018) showed a higher potency of the compound in apparent contrast with previous studies (60). In the cases here-in considered, delayed pupillary reaction, ataxia, slurred speech, bradypsychia and somnolence were commonly reported, disregarding from 5F-MDMB-PICA levels and BAC (28). This can be clearly appreciated when comparing case #22 and case #25, which had approximately the same psychomotor deficits despite very different blood concentrations. This is a further confirmation that the identification of effects of co-consumption remains extremely challenging. The evaluation of psychomotor performances should be done on a case-by-case basis and requires a multidisciplinary assessment, taking also into consideration a possible development of tolerance. Paradoxically, increased vigilance was reported in a case of 5F-MDMB-PICA and ethanol co-consumption, again in combination with altered mood, aggressiveness and psychiatric symptoms as persevering thinking (case #31) (30). This appears quite unusual since, in the majority of cases CNS depressant symptoms were the most common. Indeed, out of 464 adverse clinical effects related to SCs exposure reported to a United States Poison center, coma and respiratory depression were described, always in combination with other drugs including ethanol (15).

#### 4.2.3. Fatalities after SCs and ethanol co-consumption

It is known, and has already been described, that alcohol co-consumption is frequent in cases of death related to SCs (26, 61–65), although a recent review revisited a prevalence of 17% in this sub-setting (61). The combination of ethanol and “Spice” products might lead to death (66) even at very low concentrations (e.g., 5F-PB-22 at 0.37 ng/ml combined with a BAC of 2.60 g/kg (67)). Death might occur quickly (within 30 min from smoking, according to circumstantial data) (68), with sudden unresponsiveness or collapse (53). One case of co-consumption related death is also reported by Seywright et al. (26), but the NPS was considered not contributory. In the present review, death cases were not specifically targeted and were only included when toxicological analyses had been performed before the death. This decision was made in order to reduce biases connected to the time between death and toxicological analysis, to the sampling and analysis of post-mortem blood (which is a very different matrix from blood of living subjects), and to post-mortem redistribution (26). More specific studies might be performed in the future to understand the risk of death due to the co-consumption of ethanol and SCs.

### 4.3. Limitations

The present literature review displays several limitations. A major one resides in the wide variation in the characteristics of the included studies. Indeed, from a methodological point of view, the studies ranged from case report and case series to epidemiological (retrospective, prospective and cross-sectional) studies. Each study design has some inherent limitations and could be biased: surveys and questionnaires strongly depend on what subjects remember and what they are willing to say about a certain topic; the low sample size of case reports and case series does not allow generalization on the association between exposure and outcome. Articles were also affected by significant biases due to confounding factors not being considered, first of all the tolerance to the effects of SCs and ethanol. Biases arising from the measurement of the exposure could arise when considering all classes of NPS, not only SCs. Selection biases as well as biases arising from the measurement of the outcome of the selected studies limit the possibility of comparing the outcomes and of assessing the association between co-consumption and risks for driving. Beside limitations arising from the selected articles, some additional factors have to be mentioned: the prevalence was mathematically calculated only from the reported cases, but a publication bias should be taken into consideration; it was not assessed whether the analytical methods used covered all the analytes of interest at the timepoint of the study; a table of comparison group was not specifically included in the present review; other factors were not considered, such as the variations in substance use by country (including availability of substance, legislation, age plus other individual covariates) which could also influence the interpretation of the results.

## 5. Conclusion

The present review has shed some light on the challenges connected to the evaluation of psychomotor performances in cases of co-consumption of new psychoactive substances, particularly focusing on synthetic cannabinoids and ethanol. Although certain dose-relationship effect is known for BAC levels and driving performance, this could not be confirmed in case series involving synthetic cannabinoids. Their effects, together with the true prevalence among DUID cases, remain partially obscure. Based on the limited evidence so far reported in literature, an association between co-consumption and increased risk for road accidents was found and it seems likely that co-consumption might play a synergistic effect on the central nervous system depression, as well as on aggressiveness and mood alterations. The lack of data regarding proper sampling methods, tolerance of the involved subjects, pharmacology of compounds and their metabolites, as well as issues in detecting synthetic cannabinoids represent a challenge for a better knowledge of the risks arising from the co-consumption and for demonstrating a causality between co-consumption and driving disability.

## Author contributions

AG and RG contributed to conception and design of the study. VO organized the database. AG and VO wrote the first draft of the manuscript. FB made the critical revision of the paper. All authors contributed to manuscript revision, read, and approved the submitted version.

## Funding

This work was supported by the Department for Drug Policies, in Italy, under the project’s name “Identificazione e studio degli effetti delle NPS: Sviluppo di una multicentrica di ricerca per il potenziamento informativo dello SNAP e della base dati dell’Osservatorio nazionale sulle tossicodipendenze.”

## Conflict of interest

The authors declare that the research was conducted in the absence of any commercial or financial relationships that could be construed as a potential conflict of interest.

## Publisher’s note

All claims expressed in this article are solely those of the authors and do not necessarily represent those of their affiliated organizations, or those of the publisher, the editors and the reviewers. Any product that may be evaluated in this article, or claim that may be made by its manufacturer, is not guaranteed or endorsed by the publisher.
